# Signal Transduction in Astrocytes during Chronic or Acute Treatment with Drugs (SSRIs, Antibipolar Drugs, GABA-ergic Drugs, and Benzodiazepines) Ameliorating Mood Disorders

**DOI:** 10.1155/2014/593934

**Published:** 2014-02-24

**Authors:** Leif Hertz, Dan Song, Baoman Li, Ting Du, Junnan Xu, Li Gu, Ye Chen, Liang Peng

**Affiliations:** Department of Clinical Pharmacology, China Medical University, No. 92 Beier Road, Heping District, Shenyang, China

## Abstract

Chronic treatment with fluoxetine or other so-called serotonin-specific reuptake inhibitor antidepressants (SSRIs) or with a lithium salt “lithium”, carbamazepine, or valproic acid, the three classical antibipolar drugs, exerts a multitude of effects on astrocytes, which in turn modulate astrocyte-neuronal interactions and brain function. In the case of the SSRIs, they are to a large extent due to 5-HT_2B_-mediated upregulation and editing of genes. These alterations induce alteration in effects of cPLA_2_, GluK2, and the 5-HT_2B_ receptor, probably including increases in both glucose metabolism and glycogen turnover, which in combination have therapeutic effect on major depression. The ability of increased levels of extracellular K^+^ to increase [Ca^2+^]_*i*_ is increased as a sign of increased K^+^-induced excitability in astrocytes. Acute anxiolytic drug treatment with benzodiazepines or GABA_A_ receptor stimulation has similar glycogenolysis-enhancing effects. The antibipolar drugs induce intracellular alkalinization in astrocytes with lithium acting on one acid extruder and carbamazepine and valproic acid on a different acid extruder. They inhibit K^+^-induced and transmitter-induced increase of astrocytic [Ca^2+^]_*i*_ and thereby probably excitability. In several cases, they exert different changes in gene expression than SSRIs, determined both in cultured astrocytes and in freshly isolated astrocytes from drug-treated animals.

## 1. Introduction

Signal transduction in astrocytes is of fundamental, but largely unrecognized, importance for brain function under normal and abnormal conditions. Due to the extreme difficulty in studying specifically astrocytic signaling in the brain in vivo, many studies have been carried out in isolated preparations of astrocytes, that is, freshly isolated cells obtained directly from the brain or cultured astrocytes. Claims by the Kimelberg that astrocyte cultures are misleading [1] are unfortunately often correct. However, these researchers have unjustifiably ignored that many types of astrocyte cultures exist and that they are not similar in their characteristics. The fact that the cultures used by us are well suited to study drug-induced signaling changes is demonstrated in Table 1 (slightly modified from [2]). It shows identical changes in gene expression and editing induced by chronic treatment with drugs used to treat mood disorders (fluoxetine; carbamazepine) in cultured astrocytes and in astrocytes freshly dissociated from the brains of animals chronically treated with the same drugs [3]. Not a single gene was affected differently in the two situations. The freshly dissociated cells are probably not sufficiently intact to show the mechanisms involved in these gene changes or their functional consequences, which for this reason have been elucidated in the cultured cells. Such studies have indicated important correlations between different effects on gene expression or editing shown in Table 1 and they have enabled tentative functional interpretations.

## 2. Effects of SSRIs

### 2.1. Acute Effects

#### 2.1.1. Pathway

The prototypes of antidepressant drugs (which also have anxiolytic effect) are the serotonin-specific reuptake inhibitors (SSRIs). When fluoxetine, the first of the presently used SSRIs, was approved for clinical use in 1987, two effects of the drug had been established: inhibition of serotonin reuptake by the serotonin transporter (SERT) [6] and partial displacement of serotonin (5-HT) binding to cultured astrocytes [7], which have no SERT expression [8]. However, at that time, astrocytes were supposed to be unimportant for brain function, and inhibition of SERT has since then been regarded as the mechanism responsible for SSRIs effects. In 1987, the 5-HT_2B_ receptor was unknown, but it is now established to have the highest affinity between 5-HT receptors for SSRIs, with a K_*i*_ for displacement of serotonin binding to cultured astrocytes of 70 nM [5]. This is identical to its K_*i*_ for inhibition of serotonin uptake via the human placental SERT [9]. Moreover, all five SSRIs are equipotent in their effect on astrocytes [10]. This is a distinct difference from the large potency difference in their effect on SERT, although the therapeutic doses are of roughly comparable magnitudes, a difference, which can only partly be explained by differences in drug kinetics and protein binding.

**Figure 1:**
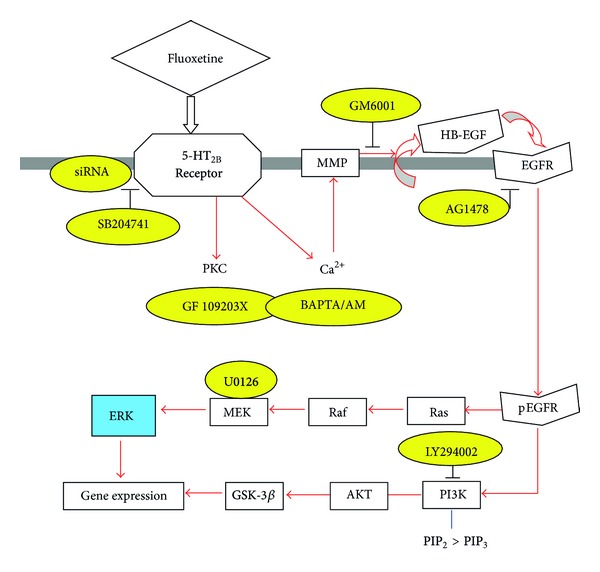
Schematic illustration of pathways leading to stimulation of ERK and AKT phosphorylation by fluoxetine in astrocytes. Fluoxetine binds to 5-HT_2B_ receptors. The activation of the receptors in turn induces an enhancement of protein kinase C (PKC) activity and of intracellular Ca^2+^ concentration by Ca^2+^ release from intracellular stores. The latter activates Zn-dependent metalloproteinases (MMPs) and leads to shedding of growth factor(s). The released epidermal growth factor receptor (EGFR) ligand stimulates phosphorylation of the EGFR. The downstream target of EGFR, extracellular-regulated kinase (ERK) (shown in blue) is phosphorylated via the Ras/Raf/MEK pathway, and AKT is phosphorylated via PI3K pathway. During chronic fluoxetine administration, inhibitors (shown in yellow) of the 5-HT_2B_ receptor (SB204741), or siRNA against this receptor, of PKC (GF 109293X), of intracellular Ca^2+^ homeostasis (BAPTA/AM, an intracellular Ca^2+^ chelator), of Zn-dependent metalloproteinases (GM6001), of the receptor-tyrosine kinase of the EGFR (AG1478), of ERK phosphorylation (U0126, a mitogen-activated kinase (MEK) inhibitor), or of the AKT pathway (LY294002, a PI3K inhibitor) prevent changes in gene expression and editing. PIK3 catalyzes the formation of PIP_3_ from PIP_2_, from Hertz et al. [5].

The metabolic pathway activated in cultured astrocytes by fluoxetine was first established by Li et al. [11] and an expanded version is shown in Figure 1. With increasing realization of the importance of glycogenolysis for signaling in astrocytes [12–14], it may be important that fluoxetine acutely stimulates glycogenolysis, an effect that is secondary to an increase in [Ca^2+^]_*i*_ [15]. Fluoxetine might also affect glycogen synthesis, since it stimulates the AKT pathway (see below). The involvement of 5-HT_2B_ receptor-stimulated glycogenolysis has been established during learning, where acute administration of serotonin can rescue long-term learning in a one trial aversive learning paradigm in day-old chickens under conditions when the aversive stimulus was otherwise too weak to establish more than transient long-term memory retention [16]. Fluoxetine and paroxetine have a similar effect and are equipotent, showing that the rescue was not due to inhibition of SERT, and the rescuing effect was inhibited by an inhibitor of glycogenolysis (Gibbs and Hertz, submitted, Frontiers in Pharmacology (Neuropharmacology) after invitation to Research Topic).

In accordance with Figure 1, fluoxetine acutely phosphorylates AKT in cultured astrocytes [5]. AKT-mediated phosphorylation of glycogen synthase kinase-3*β* (GSK-3*β*) inhibits its enzymatic activity. AKT phosphorylation may stimulate glycogen synthesis, since phosphorylation and activation of glycogen synthase by GSK-3 decreases the activity of glycogen synthase [17]. It is consistent with these observations that Li et al. [18], from the Jope group, found in whole brain that fluoxetine administration increases the levels of phosphorylated GSK3*β*. Later, Beurel et al. [19] from the same group confirmed that fluoxetine rapidly and robustly increases serine phosphorylation of GSK3 but that these responses in young mice are blunted or absent. This is consistent with an effect on astrocytes, since these glial cells are generated postnatally [20]. An increased glycogen synthesis following 5-HT_2B_ receptor activation has been directly shown in hepatocytes, whereas agonists of 5-HT_1_ and 5-HT_2A_ receptors had the opposite effect [21].

### 2.2. Chronic Effects

#### 2.2.1. Affected Genes

The effects of SSRIs that are important in connection with major depression are the chronic effects, since the effect of clinical treatment takes several weeks to appear. To-date, all gene effects caused by chronic treatment with fluoxetine (the only SSRI studied on astrocytes in vivo) after mice had been treated for 14 days with i.p. injection of fluoxetine (10 mg/kg per day) have been identical to those in cultured astrocytes chronically treated with fluoxetine or other SSRIs, as was shown in Table 1. They have also been similar to those found by other authors or ourselves in total brain with the exception of gene expression of sPLA_2_ and GluK4, genes that were upregulated in neurons of the treated animals and accordingly also in whole brain [22]. Anhedonia, one of the components of major depression, caused oppositely directed changes in expression of some of the same genes [22]. Other gene expression changes established in the cultured cells did not occur, but it should be remembered that anhedonia is only one component of major depression. Fluoxetine can reverse both the anhedonia and the gene expression changes (B. Li and L. Peng's unpublished results).

#### 2.2.2. ADAR2

The upregulation of ADAR2 by chronic treatment with fluoxetine (Table 1) depends on 5-HT_2B_ receptor stimulation, since the 150–200% increase of mRNA and protein expression of ADAR2 was prevented in astrocytes treated with 5-HT_2B_ receptor siRNA [23]. In contrast to the upregulation of cPLA_2_ (see below), it is not known if additional steps of the fluoxetine-mediated pathways are also required. ADARs constitute a family of adenosine deaminases catalyzing deamination of adenosine to inosine in double-stranded regions of mRNAs, thus changing the translated protein sequence, since inosine is read by the cells as guanosine [24]. There are three members in the ADAR family: ADAR1, ADAR2, and ADAR3 [25]. All three types of ADAR are expressed in the brain [26]. ADAR2 upregulation in fluoxetine-treated mice was specific for this subtype, observed only in astrocytes and occurred within 3 days [23]. In the brain ADAR2 has been shown in hippocampal pyramidal neurons and cerebellar Purkinje cells and Bergmann glial cells, with less expression of ADAR1 and ADAR3 [27]. ADAR2 and probably also its upregulation are essential for the editing changes shown in Table 1; this has been directly demonstrated in cultured astrocytes by the use of cells treated with siRNA against ADAR2 [23].

#### 2.2.3. cPLA_**2**_

Astrocytes are among the cells that express calcium-dependent phospholipase 2 (cPLA_2_) [27, 28], and in the brain in vivo, they may even be enriched in this phospholipase [29–31]. Its activation specifically releases arachidonic acid from the *sn*-2 position of membrane-bound phospholipid substrate in neural preparations [32–35], including glioma cells [36]. Arachidonic acid strongly stimulates glucose metabolism in cultured astrocytes [37]. So does treatment with 10 *μ*M fluoxetine for 24 h, which might have sufficed to induce an increase in cPLA_2_ [38], whereas acute exposure of astrocyte cultures to the same concentration of fluoxetine has no corresponding effect (L. Peng and L. Hertz's unpublished experiments). Arachidonic acid also stimulates glycogenolysis [39, 40].

Rapoport and coworkers [41–43] showed that chronic administration of fluoxetine leads to stimulation and enhanced mRNA and protein expression in rat brain of cPLA_2_, but not of the two other phospholipases A_2_ (secretory PLA_2_ (sPLA_2_) and intracellular PLA_2_ (iPLA_2_)). Li et al. [28] confirmed a slow and selective upregulation of mRNA and protein expression of cPLA_2a_, the major isoform of cPLA_2_, in mouse astrocytes in primary cultures during chronic incubation with 1 or 10 *μ*M fluoxetine. The upregulation was abrogated by the 5-HT_2B_ receptor antagonist SB 204741, the metalloproteinase inhibitor GM6001, and the inhibitor of EGF receptor tyrosine phosphorylation AG1478 and by U0126, the inhibitor of ERK_1/2_ phosphorylation. These are all inhibitors of the signaling pathway that was shown in Figure 1 for fluoxetine, showing that upregulation of mRNA and protein of cPLA_2_ were inhibited by the same drugs that acutely inhibit ERK_1/2_ phosphorylation and by inhibition of the phosphorylation itself. As was shown in Table 1, upregulation, specifically of cPLA_2a_, has also been found in freshly dissociated astrocytes isolated by FACS after 2-week treatment of rats with fluoxetine, whereas no corresponding effect was found in neurons [22]. This finding strongly suggests that the enhanced cPLA_2_ activity demonstrated in whole brain after chronic fluoxetine treatment [42] selectively occurs in astrocytes.

**Figure 2:**
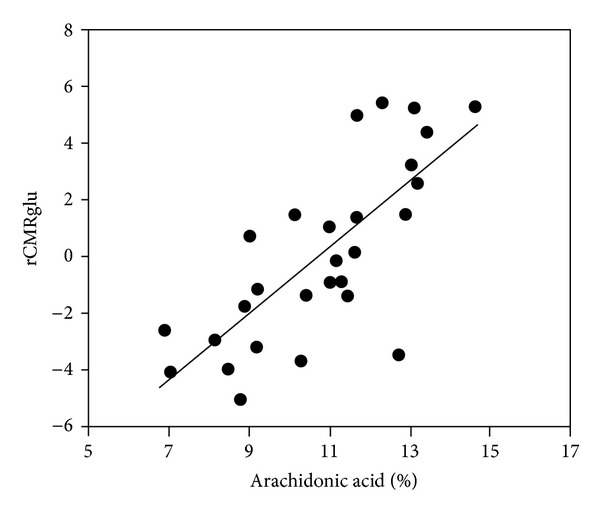
Correlation between cerebral metabolic rates of glucose metabolism and plasma arachidonic acid levels. Cerebral metabolic rates of glucose metabolism (rCMRglu) measured by NMR in vivo are shown to be correlated with plasma arachidonic acid, expressed as a percentage of total phospholipid polyunsaturated fatty acids. The correlation is statistically significant (*P* < 0.0005), from Elizabeth Sublette et al. [52].

The stimulation of glucose metabolism may be important in the pathophysiology of depressive illness and its pharmacological treatment. Glucose metabolism in brain is reduced in many regions, primarily in the frontotemporal parts, in patients suffering from unipolar depression [44–47], with a correlation between the degree of hypometabolism and severity of the illness [48], and normalization following treatment with an SSRI [49–51].  Figure 2 shows that arachidonic acid may play a role in determining rates of cerebral glucose metabolism as seen from a rectilinear correlation in depressed patients between plasma concentration of arachidonic acid and rate of cerebral glucose utilization in one of the regions affected metabolically by depression [52]. Moreover, genetic associations are found between cPLA_2_ and major depression [53, 54]. Depending on the site(s) of impairment of glucose metabolism, glycogen metabolism could also be affected. However, the effect of arachidonic acid is not necessarily exerted on glucose metabolism itself but might also be exerted on energy-requiring reactions. The importance of mitochondrial abnormalities in major depression is shown by a significantly reduced number of mitochondria, but larger mean mitochondrial volume, in hippocampus in rats sensitive to stress-induced depression than in control rats [55]. Following treatment with the antidepressant imipramine, a significant increase in the number of mitochondria occurred in the stress-sensitive group. Clinical studies also suggest that psychiatric features can be prominent features of mitochondrial disorders, but additional methodologically rigorous and adequately powered studies are needed before definitive conclusions can be drawn [56].

Arachidonic acid metabolites, including prostaglandins, may also exert other beneficial effects important for the amelioration of depression [57]. Prostaglandin synthesis inhibitors in doses used to treat pain [58, 59] may cause fear, agitation, and affective lability, and one euthymic bipolar patient repeatedly developed depression during such exposure [60]. Thus, the prostaglandins are likely to exert oppositely directed effects.

#### 2.2.4. Kainate Receptors

**Figure 3:**
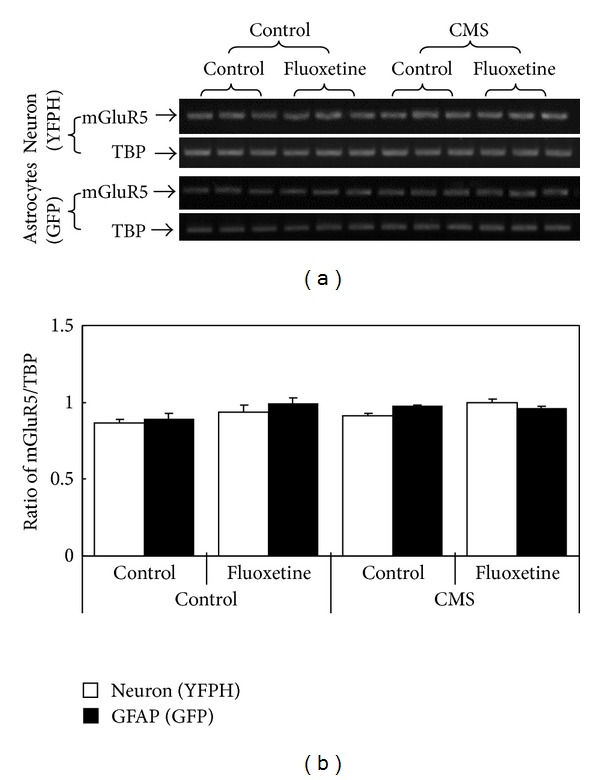
mRNA of mGluR5 is not upregulated in either astrocytes or neurons freshly isolated from animals treated with fluoxetine (10 mg fluoxetine hydrochloride/kg, i.p.) for 14 days. In both cases, astrocytes had been stained with GFP and neurons with YPHF in transgenic animals and they were separated after cell dissociation by means of the different fluorescent signals. (a) Blot showing mGluR5 RNA measured by reverse transcription polymerase chain reaction (RT-PCR) in all experiments together with that of TATA-binding protein (TBP) used as housekeeping gene (as a further check for application of similar amounts of total mRNA). mGluR5 PCR product is 513 bp. Primer sequence for mGlur5 is FWD: 5′GTCTCCTGATGTCAAGTGGTT3′; REV: 5′GGACCACACTTCATCATCATC3′. (b) mGlu5R/TBP expression ratio is virtually unaffected by fluoxetine treatment regardless of whether normal mice (left part of (b)) or mice that showed some signs of depression after exposure to chronic mild stress (CMS) (right part of (b)) were studied. Unpublished experiments by B. Li and L. Peng, using methodology similar to that used by Li et al. [22].

The only glutamate receptors shown in Table 1 are the kainate receptors GluK, among which GluK2 was upregulated and edited in astrocytes, whereas GluK4 is upregulated, but not edited, in neurons following chronic fluoxetine treatment [22]. mRNA expression analysis has demonstrated that the human GluK2c splice variant in brain is mainly expressed in nonneuronal cells and barely expressed in neurons [61]. GluK2 can operate not only in an inotropic, but also in a metabotropic mode [62]. Three positions can be edited in the genome-encoded GluK2 mRNA at 3 sites, the I/V site, the Y/C site, and the Q/R site. The editing was increased at all three sites by chronic treatment with fluoxetine [22]. This observation is consistent with another previous observation by the Barbon group that editing of the Q/R site in intact brain is slightly decreased by chronic fluoxetine treatment [63]. In most cases, inclusion of subunits containing the edited R form of the Q/R site lowers Ca^2+^ permeability [64], and in fluoxetine-treated cultures, a normally occurring increase in free cytosolic Ca^2+^ concentration of 300–400% of control value in response to 100 *μ*M glutamate was abolished by the fluoxetine treatment [23]. This increase is evoked by the GluK2 receptor operating in its metabotropic manner [5]. Glutamate-mediated inhibition of a slow neuronal afterhyperpolarization current I (sAHP) is blocked by kainate receptor(s) in the metabotropic mode [65], and inhibition of sAHP might be secondary to transmitter effects on astrocytes [66]. Inhibition of a slow neuronal afterhyperpolarization by glutamate would contribute to the importance of acute effects of glutamate in learning [16] and could also have a bearing on the mechanism by which GluK2 upregulation and editing may be therapeutically beneficial in major depression. Nevertheless, it is often assumed that increase in [Ca^2+^]_*i*_ in astrocytes is mediated by the metabotropic glutamate receptor mGluR5, but this is only in immature brain [67]. Moreover, mGluR5 is at best minimally upregulated in either astrocytes or neurons after chronic in vivo treatment of adult mice with fluoxetine (Figure 3). Mice in which the GluK2 receptor is knocked-out exhibit less anxious or more risk-taking type behavior and less manifestation of despair [68]. Obsessive-compulsive disorder is also genetically linked to abnormalities in *Grik2*, the gene coding for GluK2 [69, 70].

The role of glutamate in major depression and its drug treatment has repeatedly been discussed [5, 22, 71, 72], and some concepts are continuously changing. Hertz et al. [5] reviewed synaptic potentiation by glutamatergic stimulation of astrocytes, a topic which has been further mathematically analyzed by Tewari and Majumdar [73]. Correlations between drug effects on major depression and on glutamatergic activities have attracted special interest in connection with the rapid but short-lasting therapeutic effects of ketamine and riluzole in depressed patients. Recently, the effects of riluzole and of ketamine have been reviewed by Murrough and colleagues, Lapidus et al. [74] mainly discuss neuronal effects, although disregarding the GluK4 receptor (which according to Table 1 becomes upregulated in neurons after chronic fluoxetine treatment) but mentioning mGluRs (of which we found at least mGluR5 to be virtually unaffected by fluoxetine treatment). Murrough et al. [75] note very interesting correlations between ketamine, major depression, and cognitive function.

#### 2.2.5. The 5-HT_2B_ Receptor

**Figure 4:**
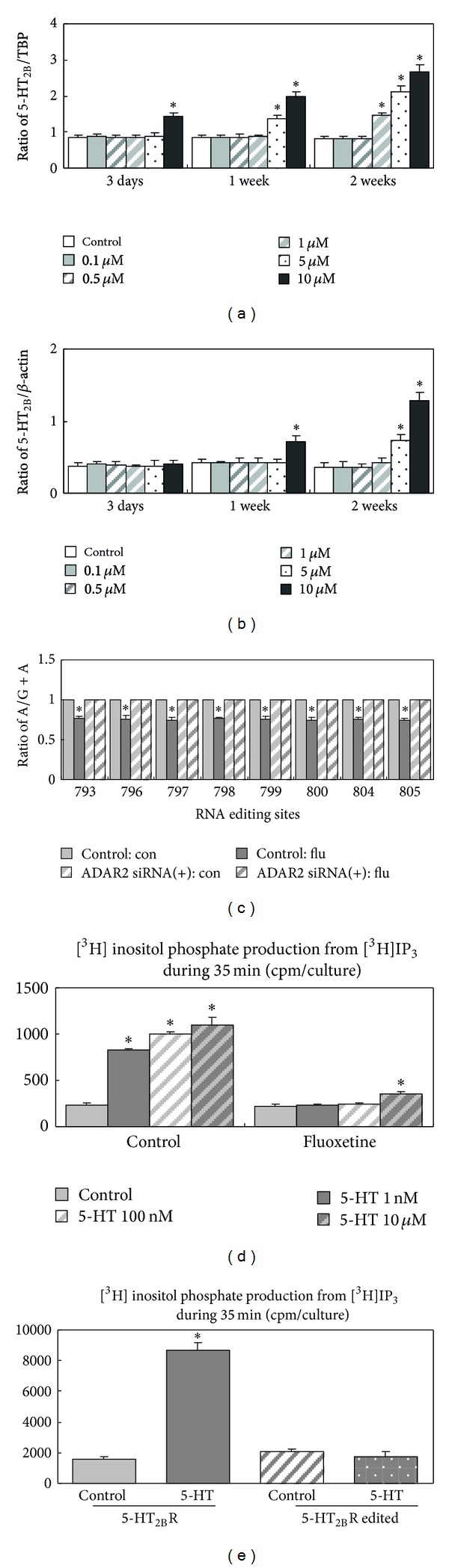
((a), (b)) Time course for upregulation of 5-HT_2B_ receptor mRNA (a) and protein (b) during treatment of cultured mouse astrocytes with different concentrations of fluoxetine. (c) Editing of 5-HT_2B_ receptor after 3 days of treatment with 10 *μ*M fluoxetine. ((d), (e)) Reduction of effect of 5-HT_2B_ receptor stimulation after downregulation of cultured astrocytes and transfected COS-7 cells with 10 *μ*M fluoxetine for 7 days. Unpublished experiments by B. Li and L. Peng. Methodologies for (a)–(c) were as in Li et al. [22]. Response of the receptor to serotonin was measured as increase in the ability of serotonin to evoke release of ^3^H-inositol phosphate (IP) from labeled IP_3_ in cultured astrocytes (d) and in cos-7 cells infected with receptor plasmids of either normal 5-HT_2B_ receptors or receptors with 8 RNA sites RNA (e). Unpublished experiments by B. Li and L. Peng.

Unpublished experiments in cultured astrocytes by Li et al. have shown that upregulation of the 5-HT_2B_ receptor itself was specific for this 5-HT_2_ receptor, since neither 5-HT_2A_ nor 5-HT_2c_ receptors were upregulated. The lack of upregulation of the latter 5-HT_2_ receptor was confirmed in freshly isolated astrocytes from fluoxetine-treated mice [22] as shown in Table 1. In contrast to the changes in gene expression of ADAR, cPLA_2_ and GluK2 described above and those in Ca^2+^ homeostasis to be discussed below, that of the 5-HT_2B_ receptor occurred very slowly (Figures 4(a) and 4(b)), but as usual with the latency depending upon the fluoxetine concentration. 5-HT_2B_ protein was not upregulated after 3 days, even at the highest fluoxetine concentration, whereas mRNA expression occurred somewhat faster (Figures 4(a) and 4(b)). In contrast editing of the 5-HT_2B_ receptor (Figure 4(c)) was obvious after 3 days of treatment, and after 7 days the edited receptor no longer responded to serotonin by an increase in activity measured as the ability of serotonin to evoke release of ^3^H-inositol phosphate (IP) from labeled IP_3_ (Figure 4(d)) or phosphorylation of ERK_1/2_ (not shown). To ascertain that the inhibition of 5-HT_2B_ receptor activity was a direct result of receptor editing and not due to other effects by chronic fluoxetine administration, COS-7 cells were infected with receptor plasmids of either normal 5-HT_2B_ receptors or receptors with 8 RNA sites RNA edited, and a similar inhibition was shown (Figure 4(e)).

#### 2.2.6. Ca^2+^ Homeostasis

In contrast to the ability of fluoxetine (and many transmitters) to acutely cause an increase in [Ca^2+^]_*i*_, chronic treatment with fluoxetine rapidly abolishes or reduces transmitter and fluoxetine-induced [Ca^2+^]_*i*_ increase [23]. However, a corresponding increase by elevation of extracellular concentrations of K^+^ above 15 mM [76] is *not* reduced, but rather increased, by chronic treatment with fluoxetine [4, 23]. The reason for this is a fluoxetine-mediated upregulation of the L-channel gene Ca_v_1.2, shown both in cultured cells and in astrocytes freshly obtained from fluoxetine-treated animals (Table 1). This may compensate for a downregulation of capacitative Ca^2+^ uptake via store-operated channels, Socs [23], as will be described below. In contrast, the Ca_v_1.3 gene, which shows higher expression in freshly isolated astrocytes than in corresponding neurons, is unaffected by treatment of mice with fluoxetine for 2 weeks [4].

**Figure 5:**
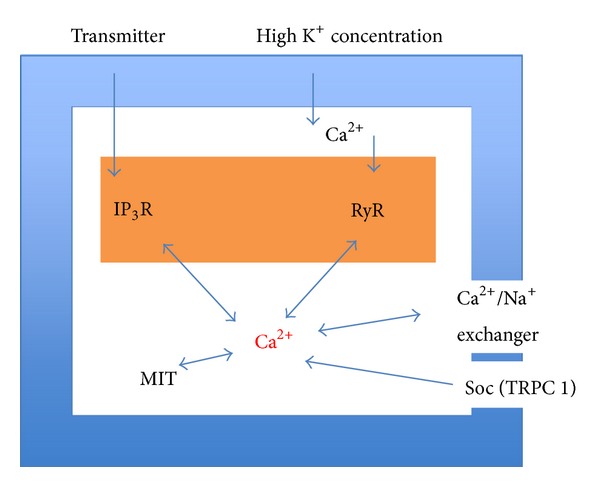
Aspects of Ca^2+^ homeostasis in astrocytes discussed in this paper. Many transmitters increase [Ca^2+^] in astrocytes by triggering release of intracellularly bound Ca^2+^ (brown) by stimulation of inositol trisphosphate (IP_3_) receptors. Highly elevated extracellular K^+^ concentrations (≥15 mM) cause L-channel-mediated Ca^2+^ entry, and additional Ca^2+^ is released by stimulation of Ca^2+^-activated ryanodine receptors (RyR). Free intracellular Ca^2+^ (red) is accumulated by ER or mitochondria or leaves the cell via a Ca^2+^/Na^+^ exchanger. This creates a need for Ca^2+^ entry via store-operated channels (Socs), of which TRPC1 is an important component in astrocytes.

Socs are very important for regulation of the amount of Ca^2+^ stored in the endoplasmic reticulum (ER) and thus the amount of Ca^2+^ released by activation of inositol trisphosphate (IP_3_) receptors (IP_3_R) or ryanodine receptors (RyR) (Figure 5). After IP_3_R- or RyR-mediated unloading of ER-bound Ca^2+^, some Ca^2+^ may enter mitochondria [77], where it exerts a stimulatory effect on several tricarboxylic acid enzymes [78], and some Ca^2+^ exits via the Na^+^/Ca^2+^ exchanger. Accordingly, the cell suffers loss of intracellular Ca^2+^, which under normal conditions is compensated for by capacitative Ca^2+^ uptake via Socs. A major component of Socs is the transient receptor potential channel (TRPC) protein TRPC1 [79]. In cells in which TRPC1 had been knocked down by treatment with antisense oligonucleotides TRPC1 antibody the capacitative Ca^2+^ uptake is also greatly reduced. The same occurs after short-lasting chronic treatment with fluoxetine and many other drugs (see below) and reduces or abrogates the ability of transmitters to increase astrocytic [Ca^2+^]_*i*_ [80]. Thus, treatment with SSRIs inhibits the ability of transmitters, at least temporarily (see below), but on account of the upregulation of Ca_v_1.2, not that of elevated K^+^ concentrations to increase astrocytic [Ca^2+^]_*i*_. This is consistent with the increased ability of elevated extracellular K^+^ concentration to increase [Ca^2+^]_*i*_ reported in [4, 23], and it is a prerequisite for transmitter effects, for example, on glycogenolysis.

#### 2.2.7. Glycogenolysis and Glycogen Synthesis

**Figure 6:**
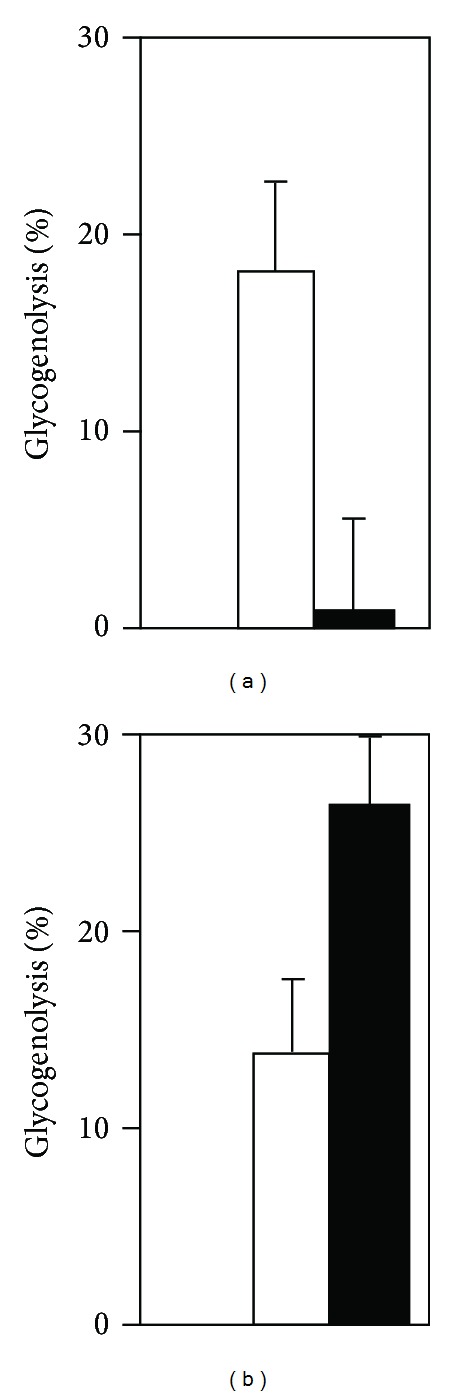
Effects on glycogenolysis (as percent of total glycogen) by 10 min acute exposure to 10 *μ*M fluoxetine in cultured mouse astrocytes chronically treated (filled columns) with 10 *μ*M fluoxetine for either 1 week (a) or for 2-3 weeks (b), compared to the effect of acute fluoxetine administration to similar untreated cultures from the same batches measured in the same experiments (open cloumns). In both (a) and (b) the treatment effect is significant (*P* < 0.05 or better). Glycogenolytic rates in (a) and (b) for the treated cultures were different at *P* < 0.0005, whereas there was no significant difference in the untreated cultures, from Kong et al. [8].

An increase in [Ca^2+^]_*i*_ is a prerequisite for stimulation of glycogenolysis, which is consistent with the reduced or abrogated ability of fluoxetine to increase [Ca^2+^]_*i*_ after 1 week of treatment with 10 *μ*M fluoxetine. However, after treatment with this concentration for 2-3 weeks acute administration of fluoxetine causes a larger increase in glycogenolysis than in control cultures [8] as shown in Figure 6. This is a very long treatment with a very high concentration, and it cannot be excluded that this finding is an artifact. However, another possibility is that it is a consequence of the late upregulation of the 5-HT_2B_ receptor. If this is the case, the inability of transmitters to increase [Ca^2+^]_*i*_ *might* also be reversed after longer treatment. However, the effects of the gene changes of ADAR2, cPLA_2_, and GluK2 are probably not further altered.

**Figure 7:**
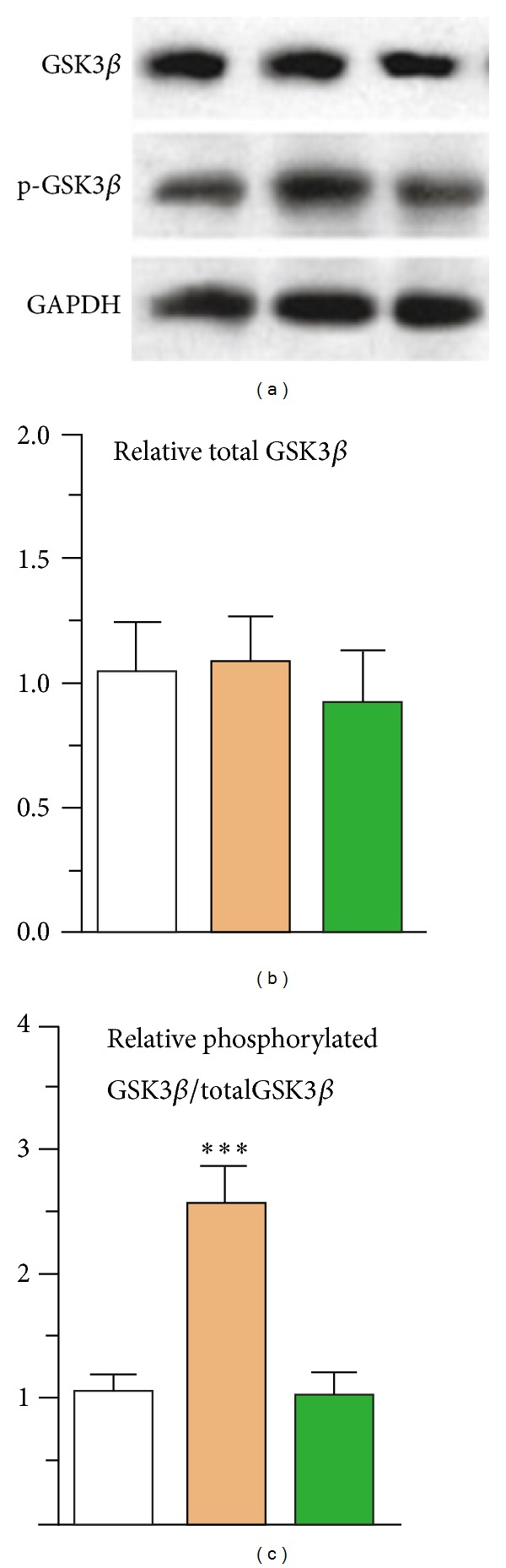
Treatment with citalopram (15 mg/kg) for 14 days (brown columns) has no effect on total GSK3*β* expression in hippocampus (a,b), compared to that in untreated control animals (white columns), but dramatically increases its phosphorylation (c). This effect is abolished by sulindac, an inhibitor of the stimulated pathway (green columns), from Liu et al. [82], with permission.

AKT phosphorylation by serotonin or 10 *μ*M fluoxetine is also impaired or abolished after 3 days of treatment with 10 *μ*M fluoxetine (B. Li and L. Peng's unpublished experiments) but may similarly recover with an increased response after 2 weeks [5]. Since GSK3*β* is phosphorylated by AKT (Figure 1), this might imply that glycogen synthesis is also increased after treatment with fluoxetine for a sufficiently long period. The importance of GSK3*β* is shown by observations that reduced immobility in the forced swim test (a sign a fear reduction) occur after administration of GSK3 inhibitors to normal animals and in GSK3*β* haploinsufficient mice and that a selective GSK3*β* inhibitor can alter serotonin-associated behavioral phenotypes in tests evaluating 5-HT-related antidepressant and anxiolytic drug effects (reviewed in [81]). More recently, Liu et al. [82] found that rats exposed to chronic mild stress showed depression-like behaviors and decreased levels of phosphorylated GSK3*β* in the hippocampus. Chronic citalopram treatment alleviated the depression-like behaviors and reversed the disruptions of the phosphorylated GSK3*β* in these animals (Figure 7). In contrast, Karege et al. [83] reported decrease in phosphorylated GSK3*β* protein and a reduced ratio between phosphorylated and total GSK3*β* in postmortem brain tissue from patients having suffered from major depression.

#### 2.2.8. Conclusion

Chronic treatment with fluoxetine, a 5-HT_2B_ agonist (confirmed by Diaz et al. [84]), exerts a multitude of effects on astrocytes, which in turn modulate astrocyte-neuronal interactions and brain function. These are to a large extent due to an upregulation and editing of genes, with rapid editing but late upregulation of the 5-HT_2B_ receptor gene itself. These alterations induce alteration in effects of cPLA_2_, GluK2, and the 5-HT_2B_ receptor, probably including increases in both glucose metabolism and glycogen turnover, which in combination have therapeutic effect on major depression. The fact that gene effects are involved probably contributes to the late manifestation of the therapeutic effect of SSRIs. Moreover, the ability of increased levels of extracellular K^+^ to increase [Ca^2+^]_*i*_ is increased as a sign of increased K^+^-induced excitability in astrocytes and therefore probably also of an enhanced ability of elevated K^+^ to stimulate glycogenolysis.

## 3. Effects of Antibipolar Drugs

### 3.1. Effects Shared by Different Antibipolar Drugs

The three classical drugs that have effect against bipolar disorder, and especially mania, are the lithium ion “lithium”, carbamazepine, and valproic acid. A common feature of these three drugs is that they, like SSRIs, must be administered for a couple of weeks for the therapeutic action to become manifest. In studies of mechanism(s) of action for antibipolar drugs, it is therefore again important to determine effects that only appear after chronic administration. Moreover, the mechanisms of drug action may with advantage be elucidated by studying shared effects of all three classical antibipolar drugs.

**Figure 8:**
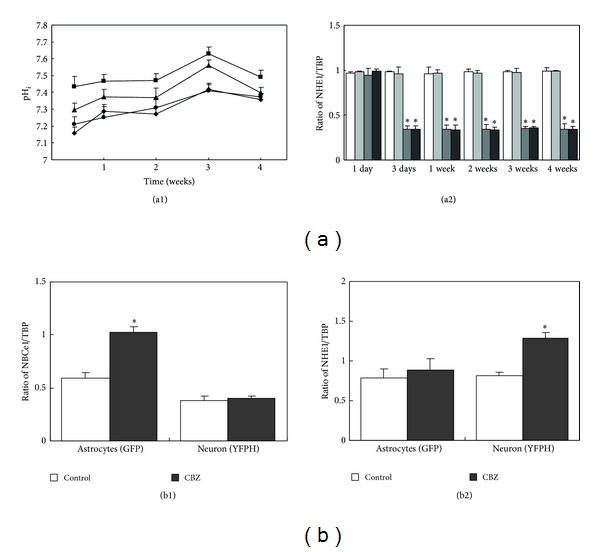
(a1) Effects of chronic treatment with different concentrations of lithium on intracellular pH measured as a function of the length of the treatment and the lithium concentration (diamonds: control; circles 0.5 mM Li^+^; triangles 1 mM Li^+^; or squares 2 mM Li^+^). (a2) mRNA expression of NHE1 in control cultures (white); cultures treated with 0.5 mM Li^+^ (light gray); 1 mM Li^+^ (darker gray) or 2 mM Li^+^ (black). *indicates statistically significant difference (*P* < 0.05 from control cultures and cultures treated with 0.5 mm Li^+^ for the same length of time. (b) mRNA expression of the acid extruders NBCe1 and NHE1 in astrocytes and neurons from astrocytes, obtained from untreated animals or animals treated for 14 days with carbamazepine (CBZ). In both cases, astrocytes had been stained with GFP and neurons with YPHF in transgenic animals, and they were separated after cell dissociation by means of the different fluorescent signals. The size of the PCR products of NBCe1 is 298 bp, of NHE1 is 422 bp, and of TBP is 236 bp. The primers for NBCe1 were (FWD) 5′CTCACTTCTCCTGTGCTTGCCT3′ and (REV) 5′GTGGTTGGAAAATAGCGGCTGG3′, and those for NHE1 and TBP the same as used by Song et al. [85]. (a1) and (a2) from Song et al. [86], ((b1) and (b2)) Unpublished experiments by B. Li, D. Song, and L. Peng, using methodology similar to that used by Li et al. [22].

A common effect of chronic treatment with these otherwise very different drugs is a gradual development of intracellular alkalinization in astrocytes, caused by stimulation of acid extruders. Lithium [85] increases intracellular pH (Figure 8(a1)) by directly stimulating the Na^+^/H^+^ exchanger NHE1, which leads to a compensatory decrease in its gene expression (Figure 8(a2)). Carbamazepine and valproic acid upregulate the expression and thus the function of the Na^+^/bicarbonate cotransporter NBCe1 [85, 86, 89] in astrocytes but not in neurons, as seen from Figure 8(b1). However, the carbamazepine treatment increases NHE1 expression in neurons, but not in astrocytes Figure 8(b2). It is likely that pH becomes increased in extracellular fluid as a result of the increased astrocytic NBCe1 function induced by carbamazepine. The neuronal increase in NHE1 expression may therefore be a defense against the development of a resulting neuronal acidosis. The intracellular astrocytic alkalinization may be the cause of an upregulation of cPLA_2_ in astrocytes shown in Table 1, since the activity of this enzyme increases with rising pH [90]. This effect is similar to that seen after chronic fluoxetine administration and may have similar consequences, including stimulation of glucose metabolism. However, cPLA_2_ is downregulated in neurons [86] and in whole brain [91] after carbamazepine treatment, which has contributed to the probably exaggerated concept of a role in neuroinflammation in bipolar disorder [57]. In contrast to the effect of fluoxetine, GluK2 expression is downregulated. Some potential effects of this downregulation are discussed by Song et al. [86], and it would be important to know the detailed differences between the consequences of the downregulated GluK2 function and the function of the upregulated, but edited Gluk2 observed after chronic treatment with fluoxetine. The most important consequence of the intracellular alkalosis is probably the effect it has on *myo*-inositol uptake and thereby on phosphatidylinositide signaling. Moreover, antibipolar drugs have effects on ion homeostasis: Na^+^ and K^+^ by altering expression of Na^+^, K^+^-ATPase subtype expression, and Ca^2+^ by downregulating TRPC1, apparently with no concomitant upregulation of Ca_v_1.2, since K^+^ effects are also reduced or abrogated as shown below.

### 3.2. Inositol Uptake and Metabolism: Transporters and pH Effects

#### 3.2.1. Depletion of *myo*-Inositol

**Figure 9:**
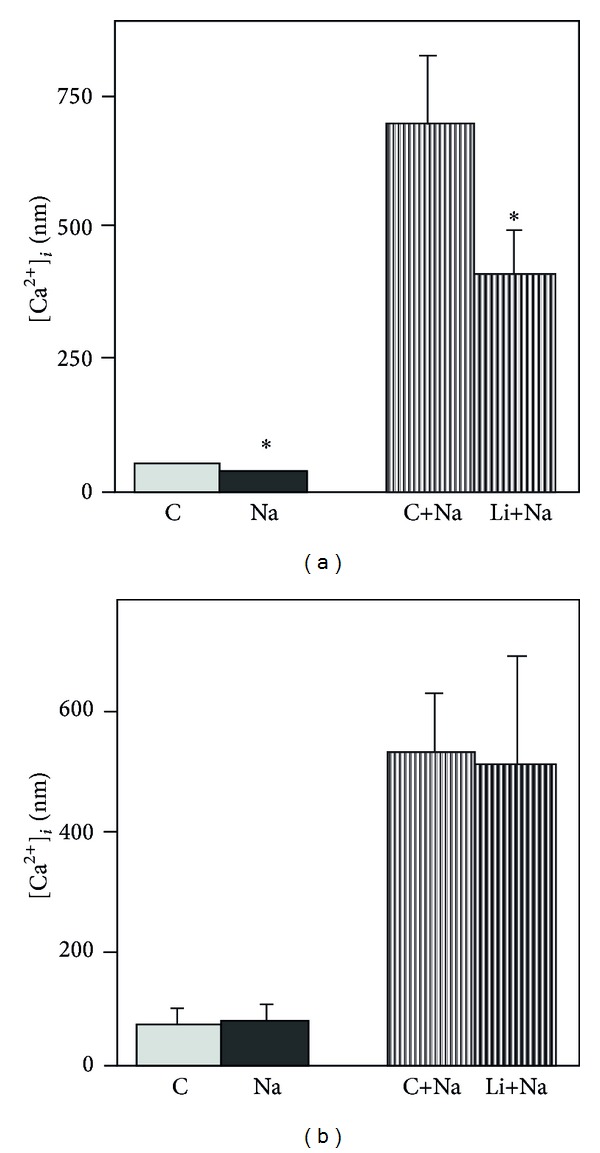
[Ca^2+^]_*i*_ in mouse astrocytes during basal conditions control (C) and during exposure to 1 *μ*M noradrenaline (Na) in untreated control cultures and in sister cultures which had been treated for either 7–14 days (a) or 30–45 min (b) with 1 mM lithium chloride (Li). The chronic lithium treatment decreased [Ca^2+^]_*i*_ significantly (*P < 0.05) both under basal condition and during exposure to noradrenaline (a), whereas the short-lasting exposure to Li^+^ had no effect, from Chen and Hertz [87].

A classical proposition, the Berridge hypothesis for lithium's therapeutic effects in bipolar disorder is that *myo*-inositol is depleted by lithium [92]. Transmitter-mediated phospholipase C (PLC) stimulation hydrolyzes phosphatidyl-4,5-bisphosphate (PIP_2_) in the cell membrane. This produces two second messengers, the cytosolic IP_3_ and the membrane-associated 1,2-diacylglycerol (DAG). IP_3_ triggers Ca^2+^ release from intracellular Ca^2+^ stores via stimulation of the IP_3_ receptor, and DAG activates protein kinase C. Subsequently, IP_3_ is stepwise dephosphorylated, eventually to *myo*-inositol by inositol phosphatases. *myo*-Inositol cannot resynthesize PIP_2_ without contribution from a DAG metabolite. Initially, DAG is converted to phosphatidic acid (PA), an ester between a diglyceride and phosphoric acid, which condenses with cytidine triphosphate to cytidine monophosphoryl-phosphatidate (CMP-PA), which combines with *myo*-inositol to form PIP_2_ via phosphatidyl-4-monophosphate (PIP) [93, 94]. According to the “Berridge hypothesis,” inhibition of the regeneration of PIP_2_ by lithium-induced reduction of inositol formation from IP decreases renewed responses to PLC-linked receptor agonists. It is consistent with this concept that a normally occurring noradrenaline-induced [Ca^2+^]_*i*_ increase in cultured astrocytes is inhibited by chronic exposure to lithium [87] (Figure 9). Moreover, in nonmedicated bipolar patients a significant *increase* in local *myo*-inositol concentration was recently shown by NMR [95]. However, the lithium effect on inositol phosphate hydrolysis to free *myo*-inositol is not shared by either carbamazepine or valproic acid. The original Berridge hypothesis is therefore insufficient to explain effects by anti-bipolar drugs, but a modified and expanded Berridge-like hypothesis will be presented as follows.

#### 3.2.2. Cellular Contents of *myo*-Inositol

The concentration of inositol in plasma is 30–60 *μ*M [96, 97], and the intracellular concentration is at a low millimolar level [98]. Accordingly, there is a steep gradient between extracellular and intracellular *myo-*inositol levels, necessitating transport mechanisms for continuous resupply of *myo*-inositol, since *myo*-inositol and many of the PIP_2_ metabolites are further degraded. This is partly effectuated by dietary uptake following a slow transfer across the blood-brain barrier [99] and partly by synthesis in brain, which occurs only in the vasculature [100]. Therefore, inhibition of cellular uptake would also deplete *myo*-inositol in astrocytes and neurons.

#### 3.2.3. Inositol Transporters

**Figure 10:**
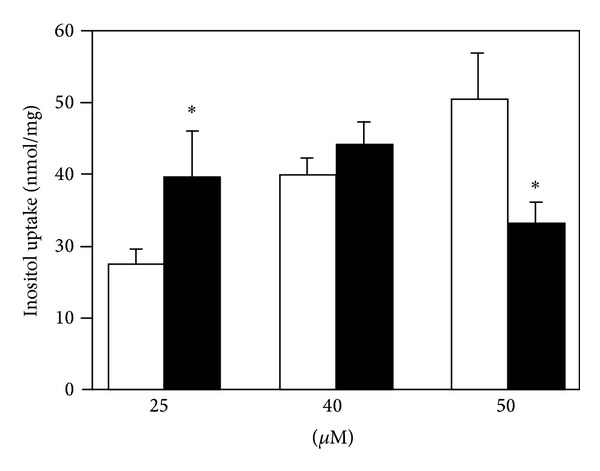
Uptake of [^3^H]*myo*-inositol at concentrations of 25, 40, and 50 *μ*M during 60 min in U251 MG astrocytoma cells treated with 1 mM lithium chloride for 2 weeks before the uptake experiment as well as during the uptake (filled columns) and in untreated control cultures (open columns). Note lithium-induced decrease of uptake at 50 *μ*M myo-inositol versus increase at 25 *μ*M. SEM values are shown by vertical bars. *Statistically significant difference between lithium-treated and control cultures (*P* < 0.05), from Wolfson et al. [88].

Wolfson et al. [101] showed that treatment of cultured mouse astrocytes with 1 mM LiCl for 8 days reduced the cellular content of *myo*-inositol after exposure to 50 *μ*M myo-inositol for 24 hours. Acute administration of lithium had no similar effect. Lubrich and van Calker [102] expanded this observation by demonstrating that chronic treatment of cultured rat astrocytes with lithium, carbamazepine, or valproic inhibited *myo*-inositol uptake. Wolfson et al. [88] confirmed this observation in cultured human astrocytoma cells at a *myo*-inositol concentration of 50 *μ*M, but at 25 *μ*M *myo*-inositol, the uptake was stimulated (Figure 10). Such an effect can be explained by the combined effects of (i) an induced alkalinization, (ii) the presence of two different *myo*-inositol transporters in astrocytes, the high-affinity Na^+^-dependent transporter (SMIT), and the lower-affinity H^+^-dependent (HMIT), and (iii) stimulation of SMIT but inhibition of HMIT at increased pH [103, 104]. We have recently confirmed that uptake of *myo*-inositol in cultured astrocytes at normal extracellular *myo*-inositol levels mainly is catalyzed by a lower-affinity, higher-capacity HMIT-mediated uptake (K_*m*_ 143 *μ*M and V_max⁡_ 358 pmol/mg protein), whereas a higher-affinity, lower-capacity SMIT-mediated uptake (K_m_ 16.7 *μ*M and V_max⁡_ 60.2 pmol/mg protein) plays a minor role [105]. Moreover, the uptake of 100 *μ*M *myo*-inositol was inhibited at increased intracellular pH, whereas that at 10 *μ*M *myo*-inositol was enhanced. The observed increase in uptake at 25 *μ*M *myo*-inositol but reduction of uptake at 50 *μ*M reported by Wolfson et al. [88] can therefore be explained by changes in relative contribution of the two *myo*-inositol transporters to total uptake on account of the induced intracellular alkalinization [85, 86]. Since di Daniel et al. [106] reported that HMIT in neurons can transport IP_3_ and Gossman and Zhao [107] and Wang et al. [108] found in the cochlea that IP_3_ can be released from nonneuronal cells through hemichannels, it cannot be excluded that astrocytically generated IP_3_ might be transferred to neurons. In that case, a decrease in astrocytic IP_3_ formation in response to antibipolar drug treatment might also affect neuronal IP_3_. Effects of transmitters operating via the protein kinase C (PKC) and the phosphatidylinositide signaling system might therefore become reduced primarily in astrocytes but possibly also in neurons by antibipolar drug treatment. Effects exerted via protein kinase A (PKA), like those of adrenaline acting on *β*-adrenergic receptors, may also be affected in astrocytes on account of a G_*s*_/G_*i*_ shift in their pathway [109]. This might include *β*-adrenergic glycogenolysis, which is dependent on the increase in [Ca^2+^]_*i*_ occurring after this shift. Since *β*-adrenergic glycogenolysis is a prerequisite for neuronal glutamatergic signaling [12, 110], reduced glycogenolysis might have considerable dampening effect on neuronal excitability. Although GSK3*β* is very often discussed in connection with this disease and its treatment, possible enhancement of glycogen synthesis seems not to have been considered or tested.

### 3.3. Ion Homeostasis

**Figure 11:**
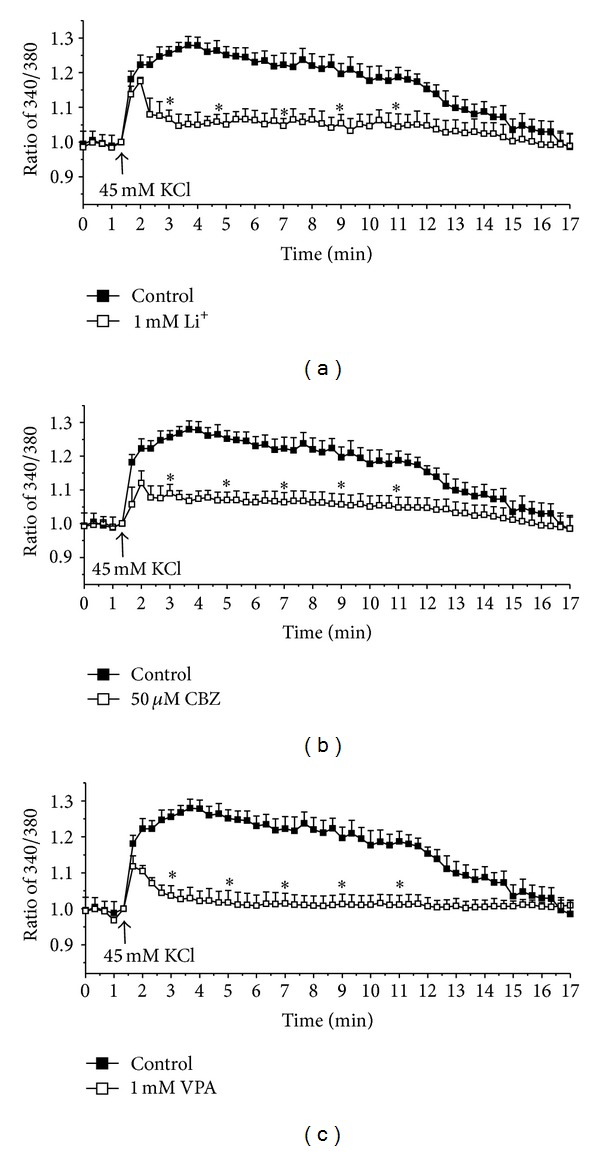
[Ca^2+^]_*i*_ in astrocytes in arbitrary units (fluorescence ratio) in response to addition of 45 mm KCl in control cultures and in cultures treated during 14 days with 1 mM lithium (a), 50 mM carbamazepine (CBZ) (b), or 1 mM valproic acid (VPA) (c), from Yan et al. [76].

Like fluoxetine chronic treatment with any of the three antibipolar drugs downregulates TRPC1 and therefore interferes with Ca^2+^ homeostasis. However, as shown in Figure 11, the ability of elevated extracellular K^+^ concentrations to increase [Ca^2+^]_*i*_ is also inhibited [76]. Thus, both transmitter-induced and depolarization-mediated changes in astrocytic [Ca^2+^]_*i*_ are reduced. This represents an important difference from astrocytes treated with an SSRI, where only the transmitter-mediated responses are inhibited [80], and the inhibition of the transmitter-induced effect *might* even be transient.

**Figure 12:**
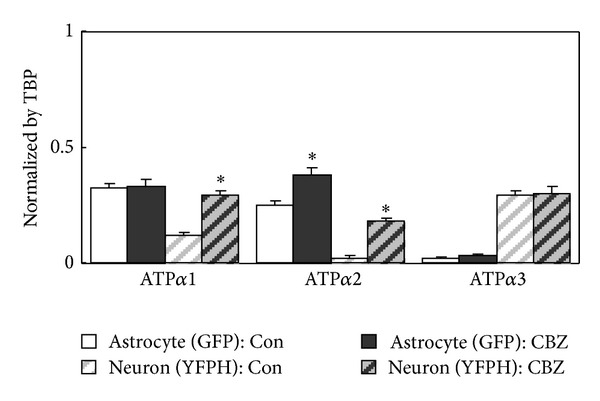
Expression of mRNA for the Na^+^, K^+^-ATPase subunits *α*1, *α*2, and *α*3, expressed as ratios between mRNA of the subtype gene and TATA-binding protein (TBP) used as a housekeeping gene in astrocytes and neurons of mice treated for 14 days with carbamazepine (25 mg/kg per day). As in Figures 3 and 8(b), the astrocytes had been stained with GFP, and the neurons with YPHF in transgenic animals, and they were separated after cell dissociation by means of the different fluorescent signals, from Li et al. [120].

An effect on TRPC1 may affect not only Ca^2+^ homeostasis, but also Na^+^ homeostasis in astrocytes, because the TRPC1 channel has equal permeability for Ca^2+^ and Na^+^ [111]. This may be of major importance in astrocytes, since these nonexcitable cells may become deficient in intracellular Na^+^ required for Na^+^, K^+^-ATPase activity [14]. Paradoxically, antibody-mediated partial inactivation of TRPC1's Ca^2+^ permeability is accompanied by an increase of the intracellular concentration of Na^+^, suggesting that the binding of the antibody to the channel decreases Ca^2+^ flux but increases Na^+^ flux [112]. Findings of decreased Na^+^, K^+^-ATPase activity in bipolar disorder are robust: (i) in brain tissue from bipolar disorder a significantly lower Na^+^, K^+^-ATPase density was found in tissues from patients having suffered from major depression or schizophrenia [113]; (ii) expression of the *α*2 gene is decreased in temporal cortex from bipolar disorder patients [114]; (iii) Na^+^-K^+^-ATPase activity is significantly reduced in erythrocytes from patients with bipolar disorder [115], and (iv) lymphoblastoid cell lines originating from bipolar patients express less Na^+^, K^+^-ATPase in response to an increase in intracellular Na^+^ concentration than similar cell lines from unaffected siblings. A possible genomic involvement of Na^+^, K^+^-ATPase dysfunction in bipolar disorder is also suggested by a genetic association between bipolar disorder and variants of the genes encoding the Na^+^,K^+^-ATPase subunits *α*1, *α*2, and *α*3 [116]. Brains in bipolar patients show increased brain acidity in vivo [117]. This might have influenced Na^+^, K^+^-ATPase activity and expression, since Deigweiher et al. [118] demonstrated that in cuttlefish gill tissues increases in water CO_2_ tension, which must decrease pH, led to a decline in Na^+^, K^+^-ATPase activity. Previously, Cummins and Hydén [119] had shown a pH maximum around 8.0 for ATP hydrolysis in microdissected astrocytes. Based on all this evidence, we investigated whether chronic treatment with carbamazepine could alter expression of Na^+^, K^+^-ATPase subtypes and of its auxiliary protein FXYD in neurons and astrocytes of normal mice [120]. Mice which coexpress one fluorescent marker with a neuron-specific gene and another marker with an astrocyte-specific gene [86] were either treated with carbamazepine for 2 weeks or received no treatment but were used as controls. From Figure 12, it can be seen that *α*3 expression was unchanged and *α*2 increased in the treated animals both in astrocytes and in neurons, where it is normally not expressed, whereas *α*1 increased only in neurons. FXYD expression was reduced by the carbamazepine treatment, but only in neurons, and *β*1 was upregulated in astrocytes, but not in neurons (results not shown). These changes should facilitate K^+^ uptake in neurons (see discussion in [120]), without compromising preferential uptake in astrocytes at increased extracellular K^+^ concentrations, a process which is important for K^+^ homeostasis of the cellular level of the brain [14]. Although an overall increase in Na^+^, K^+^-ATPase expression and presumably activity could be related to pH changes, the induced isoform expressions are not a direct consequence of the intracellular pH changes, since *α*2 was upregulated in both neurons and astrocytes, and *α*1, which shows a slight increase in activity at increased pH [121], showed increased expression in neurons but not in astrocytes. However, Na^+^, K^+^-ATPase activity, and thus expression, is regulated by a multitude of different factors, many of which may have been altered in the carbamazepine-treated mice [122].

### 3.4. Conclusion

The intracellular alkalinization induced in astrocytes by the three otherwise very dissimilar drugs lithium, carbamazepine, and valproic acid suggests that this effect is therapeutically important. If anything, this concept is strengthened by the finding that lithium acts on one acid extruder and carbamazepine and valproic acid on a different acid extruder, and by the finding of increased intracellular acidity in the brains of bipolar patients. In contrast to the ability of chronic treatment with antidepressant medication to enhance the increase of [Ca^2+^]_*i*_ and thus probably glycogenolysis by exposure to elevated extracellular K^+^ concentrations (and after sufficiently long treatment perhaps also to transmitters), chronic treatment with antibipolar drugs inhibit both K^+^-induced and transmitter-induced increase of astrocytic [Ca^2+^]_*i*_ and thereby probably excitability. The effect on astrocytic cPLA_2_ expression is similar to that after antidepressant treatment and may have similar beneficial effects, but the effect on the neuronal enzyme is the opposite. The expression of GluK2, which is upregulated (and edited) by antidepressant therapy, is downregulated, but the therapeutic consequences have not been determined with certainty. Further studies of GluK2 function in brain and drug effects on this kainate receptor under normal and pathological conditions are urgently needed. Together with the opposite effect of SSRIs and antibipolar drugs on the ability of elevated extracellular K^+^ concentrations to affect astrocytic [Ca^2+^]_*i*_, they may constitute important differences in the directions of changes caused by the antidepressant SSRIs and the mainly antimanic antibipolar drugs.

## 4. Acute Anxiolytic Drug Effects

### 4.1. GABA_**A**_ Receptor Stimulation

**Figure 13:**
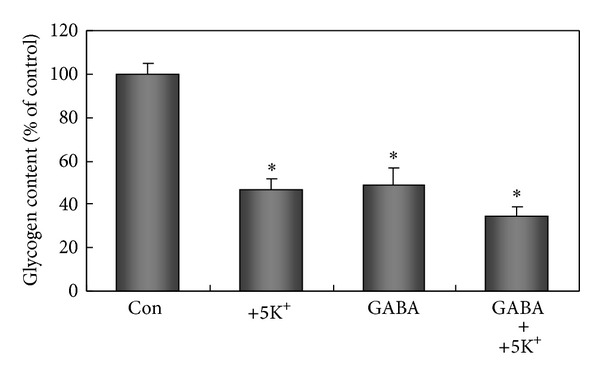
Glycogenolytic effect of GABA and/or addition of 5 mM K^+^, indicated as reduction of glycogen content, in astrocytes. Cultured astrocytes were incubated for 20 min in DMEM (containing 7.5 mM glucose) without any addition (control), addition of 5 mM K^+^ to a final extracellular concentration of 10 mM (+5 K^+^), of *γ*-aminobutyric acid (GABA) to a final concentration of 100 *μ*M (GABA), or of GABA and K^+^ (GABA plus +5 K^+^). After the incubation, the astrocytes were washed three times with ice-cold phosphate-buffered saline (PBS) and sonicated in 30 mM HCl. The suspension was used to measure nonhydrolyzed glycosyl units of glycogen. Three 50 *μ*L aliquots were sampled. In the first aliquot, 150 *μ*L of acetate buffer (0.1 M, pH 4.65) was added. In the second, 150 *μ*L of a solution containing 1% amyloglucosidase (10 mg/mL) in the acetate buffer was added in order to degrade remaining glycogen to glucose, and the mixture was incubated at room temperature for 30 min. Subsequently, the two aliquots were treated identically. Two mL of Tris-HCl buffer (0.1 M, pH 8.1) containing 3.3 mM MgCl_2_, 0.2 mM ATP, 25 *μ*g/mL NADP, 4 *μ*g/mL hexokinase, and 2 *μ*g/mL glucose-6-phosphate dehydrogenase was added to each, and the mixture was incubated at room temperature for 30 min. The fluorescence of the NADPH formed in amounts equivalent to glucose metabolized by hexokinase was then read (excitation 340 nm; emission 450 nm). The first aliquot measures the sum of glucose and glucose-6-phosphate in the tissue, whereas the second aliquot in addition to those also measures the glycosyl units from glycogen remaining in the tissue. Determination of the difference between these two aliquots provides a measurement of the amount of the latter. The third aliquot was used to measure the protein content by the Lowry method to normalize the glycogen contents (nmol) per mg protein. Average glycogen contents were indicated as percentages of those under control conditions. All values were expressed as means ± S.E.M indicated by vertical bars and were from three-five individual cultures. *Statistically significant (*P* < 0.05) difference from control. Results are unpublished experiments by J. Xu, D. Song, L. Hertz, and L. Peng.

Because of a high intracellular Cl^−^ concentration in astrocytes [123, 124], GABA_A_ receptor-mediated increase of Cl^−^ fluxes causes a depolarization, resulting in GABA_A_-induced [Ca^2+^]_*i*_ increases [124, 125] and in glycogenolysis (Figure 13). These effects are strikingly similar to those seen after chronic treatment with fluoxetine and are consistent with the observation that GABA_A_ but not GABA_B_ receptor stimulation has antianxiety-like effects in rats tested in the elevated-plus-maze [126]. Recent publications suggest that the role of GABA effects on astrocytes is currently greatly underestimated [124, 127]. This is in spite of astrocytic subunit composition of the GABA_A_ receptor indicative of an anxiolytic role.

GABA_A_ receptors are made up of *α*, *β*, and *γ* subunits, each of which can be further subdivided. In the present context, the *γ*1 subunit, which is densely expressed in basal ganglia [130] but scarcely in cortex, may be of special interest. A comparison between mRNA expression levels of different GABA_A_ receptor subunits in freshly isolated astrocytes and neurons [131] showed that >90 percent of *β*1 cortical mRNA was astrocytic, and astrocytes also accounted for ~70 percent of *α*2 expression, ~60% of *β*1, and ~40% of *α*4 (Table 2). Since limbic areas are enriched in the *γ*1 subunit, GABA_A_ receptors containing this subtype might serve affective functions [132], and the *α*2 subunit is known to mediate anxiolysis [133]. Future attempts to develop anxiolytics acting on GABA_A_ receptors [133] should include potential effects on astrocytes.

### 4.2. Effects of Benzodiazepines: Emphasis on a Membrane Receptor

It was initially believed that all anxiolytic effects of benzodiazepines were exerted on the neuronal “central” benzodiazepine receptor [134], which facilitated GABAergic transmission by binding to its receptor. Since this effect was abrogated by the benzodiazepine antagonist flumazenil, it was often concluded that all benzodiazepine effects that were inhibited by this antagonist were exerted on the “central” benzodiazepine receptor. However, the peripheral-type benzodiazepine receptor localized in mitochondria, the translocator protein (18 kDa) [135], is also important in anxiolysis [136]. It is stimulated not only by exogenously administered benzodiazepines but also by derivatives of the 86-amino acid polypeptide diazepam-binding inhibitor (DBI), which is found in brain [137]. It can be cleaved into several biologically active peptides, including the triakontatetraneuropeptide, TTN, and the octadecaneuropeptide ODN [138]. Gandolfo et al. [139] showed (i) that binding of the peripheral-type benzodiazepine receptor ligand [^3^H]Ro5-4864 to intact astrocytes cultures was displaced by TTN, whereas ODN did not compete for [^3^H]Ro5-4864 binding and (ii) that TTN provoked a concentration-dependent increase in [Ca^2+^]_*i*_ in the cultured astrocytes, which was blocked by chelation of extracellular Ca^2+^ by EGTA or blockage of Ca^2+^ channels with Ni^2+^ and significantly reduced by the L-type calcium channel blocker nifedipine. Patch-clamp studies showed that TTN induced a sustained depolarization, and the authors suggested that TTN acted through the peripheral benzodiazepine binding site and opening of Cl^−^ channels, that is, a mechanism similar to that of stimulation of GABA_A_ receptors.

It has long been known—and virtually ignored—that peripheral benzodiazepine receptors are present not only in mitochondria, but also at the plasma membrane of astrocytes [140–142]. In addition, the peripheral benzodiazepine binding site shows multiplicity and has also been demonstrated on a plasma membrane-enriched preparation from rat astrocytes [143]. Backus et al. [140] suggested the same mechanism for Ca^2+^ entry as Gandolfo et al. [139], that is, a depolarizing effect. In contrast, Bender and Hertz [141] and Zhao et al. [142], using cultures which had been differentiated by treatment with dibutyryl cyclic AMP and therefore express L-channels, suggested a direct interaction between the benzodiazepine and this channel. It seems consistent with this interpretation that midazolam had no significant effect at nonelevated extracellular K^+^ concentrations in the experiments by Zhao et al. [142]. More conclusive evidence has been reached in cardiac cells, where direct activation of the channel by the Ca^2+^ channel activator BAY K 8644 was blocked by the benzodiazepine antagonist PK11195 [144, 145]. Nevertheless, the most important finding, an increase in astrocytic [Ca^2+^]_*i*_, is identical in all these studies.

**Figure 14:**
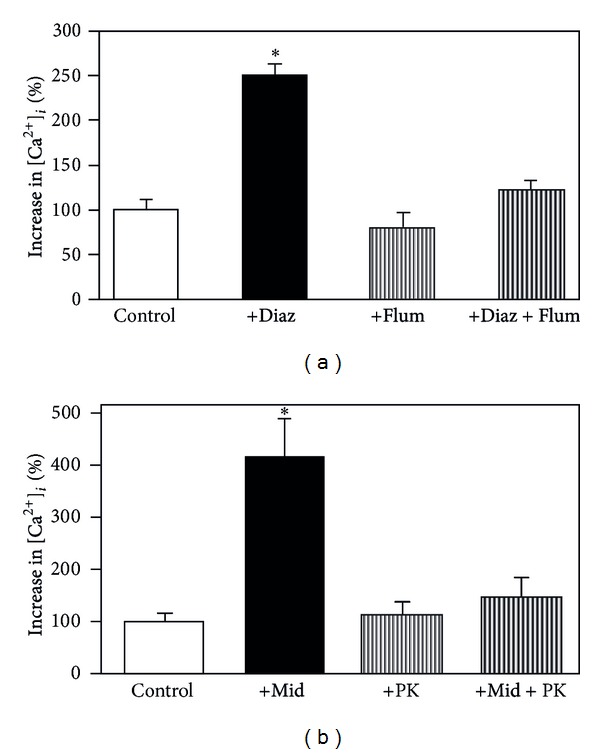
(a) Effects of 500 nM diazepam (+Diaz.), of 1 *μ*M of the “neuronal-type benzodiazepine antagonist” flumazenil (+Flum), and of diazepam plus flumazenil (+Diaz +Flum) on the increase in [Ca^2+^]_*i*_ evoked by an increase in the extracellular K^+^ concentration to 20 mM. The control value (100%) represents the increase by the elevated K^+^ concentration alone, which approximately doubled resting [Ca^2+^]_*i*_ (about 100 nM). Diazepam more than doubles the response to the increase in extracellular K^+^, and this effect is abrogated by flumazenil. The value in the presence of diazepam is statistically significantly different from all other values, none of which differs from any of the other. (b) Effects of 10 nM midazolam (+Mid.), of 1 *μ*M of the “mitochondrial benzodiazepine antagonist” PK 11195 (+PK), and of midazolam plus PK 11195 (+Mid. +PK) on the increase in [Ca^2+^]_*i*_ evoked by an increase in the extracellular K^+^ concentration to 20 mM. Midazolam almost quadruples the response to the increase in extracellular K^+^, and this effect is abrogated by PK 11195. The value in the presence of midazolam is statistically significantly different from all other values, none of which differs from any of the other, from Hertz et al. [128].

**Figure 15:**
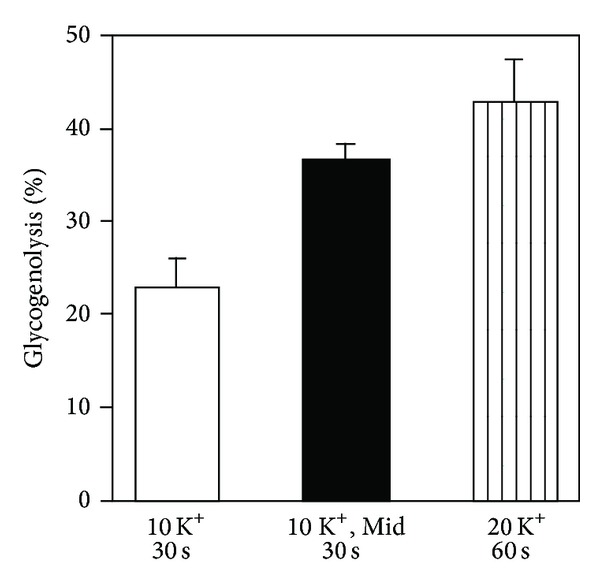
Release of previously incorporated label in glycogen during exposure to 10 mM extracellular K^+^ for 30 sec in the presence and absence of 20 nM midazolam and to 20 mM K^+^ without midazolam for 60 sec. In the presence of midazolam, glycogenolysis during exposure to 10 mM K^+^ for 30 sec is significantly increased and its magnitude becomes similar to that seen after 1 min of exposure to 20 mM K^+^ alone, from Subbarao et al. [129].

As shown in Figures 14(a) and 14(b), the benzodiazepine-mediated augmentation of the response to elevated K^+^ concentrations is selectively prevented in the presence of 1 *μ*M of the “peripheral-type” benzodiazepine antagonist PK 11195 and notably also by the supposedly “central-type” antagonist flumazenil [128, 142, 146]. The inhibition by flumazenil confirms previous observation by Backus et al. [140] and emphasizes again that inhibition of a benzodiazepine response by flumazenil does *not* necessarily indicate that the response is exerted on neuronal receptors. The dihydropyridine L-channel blocker nimodipine abolishes both the effect of elevated K^+^ and that of the benzodiazepine [142], although it should have no effect on the latter if only an L-channel-independent depolarization was involved.  Figure 15 shows that midazolam also increases the glycogenolytic effect of elevated extracellular K^+^ concentrations [129]. The high potency of benzodiazepines in increasing L-channel opening in astrocytes (500 nM diazepam and 10 nM midazolam in Figure 14) is an indication that the response may be relevant for clinically observed benzodiazepine effects, because the effective concentrations are similar to those obtained clinically [147, 148]. In contrast, stimulation of GABA-mediated Cl^−^ influx in cortical neurons by 100–1000 nM diazepam causes only a 5–40% increase [149]. Peripheral-type receptors are also present on several additional, nonneural cell types as discussed by Gandolfo et al. [139], and mature erythrocytes, which have no mitochondria, express peripheral-type benzodiazepine receptors [150]. These sites should *not* be identified as translocator proteins and have been referred to as the “Joker” receptor by Hertz et al. [128] and Hertz and Chen [146].

Yet another indication that drugs increasing [Ca^2+^]_*i*_ and causing glycogenolysis can provide acute anxiolytic effects is that acute treatment with the 5-HT_2B_ receptor agonist, BW 723C86, has been found to exert anxiolytic effects in animal experiments [151–153]. This is the same receptor as that activated by fluoxetine and other SSRIs, and acute administration of fluoxetine increases [Ca^2+^]_*i*_ and causes glycogenolysis [154]. Why some degree of anxiolysis can be obtained by acute 5-HT_2B_ activation, but the antidepressant effects require weeks of treatment and gene editing and up-regulation, remains to be shown.

### 4.3. Conclusion

Acute anxiolytic drug therapy induces Ca^2+^-dependent anxiolysis and enhances glycogenolysis evoked by exposure to elevated extracellular K^+^ concentrations, an effect which is strikingly similar to that of chronic treatment with an SSRI, which also has anxiolytic effect.

The response to GABA_A_ receptor stimulation is secondary to depolarization and that to benzodiazepines to a direct effect on the L-channel. One might wonder about the importance of L-channel opening, since it, in experiments where [K^+^]_*e*_ is the only factor changed, requires [K^+^]_*e*_ values above those usually seen in response to normal stimulation. However, it should be remembered that the [K^+^]_*e*_ is probably normally not changed alone but that the change might be accompanied by changes in transmitters, like GABA, that also contribute to the depolarization, as seen by the tendency to a more pronounced glycogenolysis in Figure 13. Accordingly, activation of Ca^2+^ uptake by elevated [K^+^]_*e*_ may occur much more frequently than should be anticipated based on the experiments in which [K^+^]_*e*_ alone is increased. This may also be of importance for the oppositely directed changes in effects of increase in [K^+^]_*e*_ after treatment with SSRIs and with antibipolar drugs.

## 5. Concluding Remarks

Astrocytic increase in [Ca^2+^]_*i*_ and in glycogenolysis after chronic treatment with SSRIs (which also have antianxiolytic effects) and during acute GABA_A_ stimulation or administration of benzodiazepines is probably important for antidepressant-anxiolytic drug effects, and astrocytic glycogenolysis is essential to support astrocytic signaling. The glycogenolytic effect is unlikely to be shared by antibipolar drugs, which all cause intracellular alkalinization in astrocytes, although by stimulation of different acid extruders. These drugs inhibit effects of both elevated K^+^ concentrations and transmitters on astrocytic [Ca^2+^]_*i*_. Carbamazepine was also shown to have important effects on Na^+^, K^+^-ATPase in both astrocytes and neurons. Both antidepressant drugs and antibipolar drugs affect GluK2, but the effects are different, and further studies of this kainate receptor are warranted in both major depression and bipolar disease. So is that of the astrocytic upregulation of cPLA_2_.

## Figures and Tables

**Table 1:** Comparison between effects on gene expression and editing of chronic treatment with the SSRI fluoxetine or the antibipolar drug carbamazepine in cultured mouse astrocytes and in astrocytes freshly isolated from drug-treated mice.

Gene	Drug	FACS, astrocytes	Culture astrocytes
ADAR2	Fluoxetine	Up	Up
5-HT_2B _receptor expression	Fluoxetine	Up	Up
5-HT_2B _editing	Fluoxetine	Up	Up
5-HT_2c_ receptor expression	Fluoxetine	Unchanged	Unchanged
cPLA_2a_	Fluoxetine	Up	Up
sPLA_2_	Fluoxetine	Unaltered	Unaltered
GluK2 expression	Fluoxetine	Up	Up
GluK2 editing	Fluoxetine	Up	Up
GluK4 expression	Fluoxetine	Unchanged	Unchanged
cfos expression	Fluoxetine	Up	Up
fosB expression	Fluoxetine	Up	Up
Ca_v_ 1.2	Fluoxetine	Up	Up
NBCe1	Carbamazepine	Up	Up
GluK2	Carbamazepine	Down	Down
cPLA_2_	Carbamazepine	Up	Up

Table 1 shows all experiments in which drug effects were compared in cultured astrocytes and in astrocytes freshly obtained by FACS as described by Lovatt et al. [3]. For FACS, astrocytes had been obtained from mice stained with GFP, based on expression of the astrocyte-specific GFAP, in transgenic animals and the stained cells were separated after cell dissociation by means of their fluorescent signal. The cultures were treated chronically with either fluoxetine or carbamazepine, and the animals had been treated chronically for a similar length of time (2 weeks). Complete agreement was found. From Peng et al. [2], with the exception of Ca_v_1.2, which is from Du et al. [4], using similar techniques.

**Table 2:** Percentage astrocytic expression (astrocytic expression/(astrocytic + neuronal expression)) of GABA_A_ receptor subunits as found by Cahoy et al. [131] in astrocytic and neuronal cell fractions obtained from murine brain*.

GABA_A_ receptor subunit	Percentage astrocytic expression
*α*1	0
*α*2	69.6 ± 3.65
*α*4	43.9 ± 2.15
*α*5	0
*β*1	61.4 ± 8.73
*β*3	0
*γ*1	92.6 ± 0.50
*γ*2	0

*No information was provided about *β*2.
